# An Analysis of Cesarean Section and Emergency Hernia Ratios as Markers of Surgical Capacity in Low-Income Countries Affected by Humanitarian Emergencies from 2008 – 2014 at Médecins sans Frontières Operations Centre Brussels Projects

**DOI:** 10.1371/currents.dis.5e30807568eaad09a3e23282ddb41da6

**Published:** 2015-03-27

**Authors:** Barclay Stewart, Evan Wong, Jessica Papillon-Smith, Miguel Antonio Trelles Centurion, Lynette Dominguez, Supongmeren Ao, Basimuoneye Kahutsi Jean-Paul, Mustafa Kamal, Rahmatullah Helmand, Aamer Naseer, Adam L. Kushner

**Affiliations:** Department of Surgery, University of Washington, Seattle, Washington, USA; Centre for Global Surgery, McGill University Health Centre, Montreal, Quebec, Canada; Surgeons OverSeas (SOS), New York, New York, USA; Department of Obstetrics and Gynecology, McGill University, Montreal, Quebec, Canada; Surgery, Anesthesia, Gynecology, and Emergency Medicine Unit, Médecins Sans Frontières, Brussels, Belgium; Surgery, Anesthesia, Gynecology, and Emergency Medicine Unit, Médecins Sans Frontières, Brussels, Belgium; Surgery, Anesthesia, Gynecology, and Emergency Medicine Unit, Médecins Sans Frontières, Brussels, Belgium; Mon General Hospital, Mon, Nagaland, India; Surgery, Anesthesia, Gynecology, and Emergency Medicine Unit, Médecins Sans Frontières, Brussels, Belgium; Referral General Hospital of Masisi, Masisi, Democratic Republic of the Congo; Surgery, Anesthesia, Gynecology, and Emergency Medicine Unit, Médecins Sans Frontières, Brussels, Belgium; Timurgara DHQ Hospital, Timurgara, Pakistan; Surgery, Anesthesia, Gynecology, and Emergency Medicine Unit, Médecins Sans Frontières, Brussels, Belgium; Ahmad Shah Baba General Hospital, Kabul, Afghanistan; Surgery, Anesthesia, Gynecology, and Emergency Medicine Unit, Médecins Sans Frontières, Brussels, Belgium; Dargai DHQ Hospital, Dargai, Pakistan; Surgeons OverSeas (SOS), New York, New York, USA; Department of International Health, Johns Hopkins Bloomberg School of Public Health, Baltimore, Maryland, USA; Department of Surgery, Columbia University, New York, New York, USA

## Abstract

Background: Surgical capacity assessments in low-income countries have demonstrated critical deficiencies. Though vital for planning capacity improvements, these assessments are resource intensive and impractical during the planning phase of a humanitarian crisis. This study aimed to determine cesarean sections to total operations performed (CSR) and emergency herniorrhaphies to all herniorrhaphies performed (EHR) ratios from Médecins Sans Frontières Operations Centre Brussels (MSF-OCB) projects and examine if these established metrics are useful proxies for surgical capacity in low-income countries affected by crisis. Methods: All procedures performed in MSF-OCB operating theatres from July 2008 through June 2014 were reviewed. Projects providing only specialty care, not fully operational or not offering elective surgeries were excluded. Annual CSRs and EHRs were calculated for each project. Their relationship was assessed with linear regression. Results: After applying the exclusion criteria, there were 47,472 cases performed at 13 sites in 8 countries. There were 13,939 CS performed (29% of total cases). Of the 4,632 herniorrhaphies performed (10% of total cases), 30% were emergency procedures. CSRs ranged from 0.06 to 0.65 and EHRs ranged from 0.03 to 1.0. Linear regression of annual ratios at each project did not demonstrate statistical evidence for the CSR to predict EHR [F(2,30)=2.34, p=0.11, R2=0.11]. The regression equation was: EHR = 0.25 + 0.52(CSR) + 0.10(reason for MSF-OCB assistance). Conclusion: Surgical humanitarian assistance projects operate in areas with critical surgical capacity deficiencies that are further disrupted by crisis. Rapid, accurate assessments of surgical capacity are necessary to plan cost- and clinically-effective humanitarian responses to baseline and acute unmet surgical needs in LICs affected by crisis. Though CSR and EHR may meet these criteria in ‘steady-state’ healthcare systems, they may not be useful during humanitarian emergencies. Further study of the relationship between direct surgical capacity improvements and these ratios is necessary to document their role in humanitarian settings.

## Introduction

Conditions requiring surgical care are responsible for more than 15% of the world’s death and disability. 1 ^,^ 2 Regrettably, this burden falls disproportionately on the lowest income countries, which are least equipped to provide timely and quality care.^2^^,^ 3 ^,^ 4 Assessments of surgical capacity in low-income countries (LICs) have demonstrated critical lack of infrastructure, personnel, equipment and supplies resulting in inordinate unmet surgical needs.^5^^,^ 6 ^,^ 7 ^,^ 8 ^,^ 9 ^,^ 10 ^,^ 11 ^,^ 12 ^,^ 13 ^,^ 14 ^,^ 15 Though vital in producing data for baseline and serial evaluations of surgical capacity, these assessments are time-consuming and resource intensive, which make them impractical during the planning and re-evaluation phases humanitarian crises.

Low-income countries generally have high birth rates and limited antenatal care resulting in the need for a large number of cesarean sections (CS).^11^ Hospitals with limited capacity in LICs report high proportions of CSs from their case volume.^5^ Hernias represent unmet surgical needs in LICs that often require emergency surgery for incarceration, strangulation or obstruction when elective herniorrhaphies are not routinely performed. In efforts to develop simpler metrics for rapid assessment of surgical capacity in LICs, several reports documented the use of the CSs to total operations performed ratio (CSR) or emergency herniorrhaphies to total herniorrhaphies performed ratio (EHR).^15^^,^ 16 ^,^ 17 A CSR >0.2 or an EHR >0.1 represents critical surgical capacity deficiencies. These reports also demonstrated that health systems with more surgical capacity have a low CSR and EHR, indicating more varied case mix and added ability to perform elective procedures. For reference, high-income countries have CSRs <0.03 and EHRs <0.08. Given CSs and herniorrhaphies are routinely logged in even the most basic surgical facilities, these easy-to-calculate metrics are readily available.^7^^,^ 18

In addition to providing surgical care for conditions attributable to crisis, humanitarian surgical assistance programs care for common surgical emergencies and elective surgical needs unmet by national healthcare systems.^19^^,^ 20 When deploying projects during the acute phase of a crisis, humanitarian organizations currently allocate resources based on extrapolated estimates of surgical need based on the country’s national income and the cause of crisis. However, baseline unmet surgical needs vary markedly across LICs.^3^^,^ 4 ^,^ 21 Unmet humanitarian assistance needs increased US$ 400 million dollars in 2013 in the face of increasing numbers of individuals affected by crisis and a growing surgical burden.^21^^,^ 22 Therefore, accurately and quickly assessing surgical needs during crisis to avoid resource mismatch has never been more important.

Médecins sans Frontières (MSF) is a humanitarian medical organization that responds to crises primarily in low-resource settings. MSF Operations Center Brussels (MSF-OCB) is one of five MSF divisions capable of providing surgical care and routinely collects data on surgical procedures performed at each project. As MSF-OCB cares for unmet surgical needs, its procedures inevitably reflect local surgical capacity. Subsequently, MSF-OCB projects provide a unique opportunity to assess potential uses of the CSR and EHR. This study aimed to determine CSRs and EHRs from MSF-OCB projects and examine how they correlate as metrics for surgical capacity in LICs affected by crisis.

## Methods

Ethics

This retrospective description of de-identified, routinely collected data satisfied criteria for MSF Ethical Review Board exemption. The Johns Hopkins Bloomberg School of Public Health Institutional Review Board provided ethical approval for secondary analyses.

Data collection

All procedures performed in an operating theatre managed by MSF-OCB worldwide are recorded using a standardized Patient Surgical Record (PSR). The PSR is transcribed monthly into a database (Excel; Microsoft, Redmond, WA) and transmitted to MSF-OCB headquarters in Brussels, Belgium. At headquarters, the Surgical, Anesthesia, Gynecology and Emergency Medicine Unit review PSRs for completeness and accuracy. Discrepancies and missing data are immediately corrected after reconciliation with respective program personnel.

Data analysis

Projects focused solely on a single condition (i.e. maternity care, obstetric fistulas, trauma during conflict, specialist missions) were excluded. In addition, projects not offering elective surgery or performing less than three operations five days per week, which was considered not fully operational, were excluded. The reason for MSF-OCB assistance for each project was characterized as natural disaster, hospital support or conflict. Programs not caring for those injured as a result of widespread conflict or natural disaster were considered to be hospital support. There is some overlap between reasons for assistance. For instance, conflict in fragile states often degenerates to protracted complex humanitarian emergencies that require hospital support despite no ongoing conflict. For the analysis, the referent reason for MSF-OCB assistance was natural disaster; the next category was hospital support and the highest category was conflict (i.e. 0=natural disaster; 1=hospital support; 2=conflict). Operations from July 2008 through June 2014 were combined and described.

In preparation for regression of CSRs and EHRs, a scatter plot revealed no obvious relationship. There were a number of potential outliers on the plot that were confirmed by casewise diagnostics; however, they represented true case ratios at each respective project; therefore, outliers were not dropped. The kernel density estimate and normal probability plot of the residuals both revealed slight non-normality, mainly in the middle range of the ratios. However, the Shapiro-Wilk test did not indicate non-normality (*p*=0.13). When the residual variances were plotted against the predicted values, the pattern widened marginally with higher predicted values. Nonetheless, White’s and Cook-Weisberg’s tests for heteroskedasticity did not provide marked evidence for non-constant residual variance (*p*=0.06 and *p*=0.14, respectively). CSRs and EHRs were neither collinear (condition number=3) nor demonstrated a departure from linearity. The Durbin-Watson and Ljung-Box Q tests did not demonstrate autocorrelation (*d*=2.04 and *p*>0.25, respectively). Since there was potential for influential outliers and slight heteroskedasticity discovered during regression diagnostics, a sensitivity analysis was performed using robust regression, quintile regression and regression with Huber-White variance-covariance estimators. These models revealed negligible differences in output. Thus, only the robust model is reported. All regression models were adjusted for reason for MSF-OCB assistance as described above. Analyses were done with Stata v13 (College Station, Texas).

## Results

A total of 96,239 operations were performed at 27 MSF-OCB sites in 15 countries between 2008 and 2014. After excluding specialist projects, projects not offering elective surgery and projects not fully operational, there were 47,472 cases performed at 13 sites in 8 countries (Table 1).

There were 13,939 CSs performed (29% of total cases). Of the 4,632 herniorrhaphies performed (10% of total cases), 30% were emergency procedures. CSRs ranged from 0.06 to 0.65 and EHRs ranged from 0.03 to 1.0. There were no clear associations with the ratios and reason for MSF-OCB assistance in each project. The ratios averaged over operational years by project are reported in Table 1.

**Table 1: Cesearean section to total operations performed ratio and emergency herniorrhaphies to all herniorrhaphies performed ration at Médecins Sans Frontières Operations Centre Brussels projects from 2008 – 2014.** 

		Proposed surgical capacity ratios	
		Cesarean section	Emergency hernia
Afghanistan	Kabul	0.50	0.62
Lashkar Gah	0.15	0.21
Central African Republic	Bangassou	0.13	1.00
Côte d'Ivoire	Abidjan	0.29	1.00
Democratic Rep. of the Congo	Lubutu	0.17	0.24
Masisi	0.44	0.39
Niangara	0.11	0.37
Haiti	Cité Soleil	0.31	0.58
Pakistan	Dargai	0.12	0.09
Timurgara	0.47	0.91
Somalia	Burao	0.33	0.72
Guri-El	0.09	1.00
South Sudan	Gogrial	0.06	1.00

Linear regression of annual ratios at each project did not demonstrate statistical evidence for the CSR to predict EHR [*F*(2,30)=2.34, *p*=0.11, *R^2^*=0.11] (Figure 1). The regression equation was: EHR = 0.25 + 0.52(CSR) + 0.10(reason for MSF-OCB assistance).

**Plot of annual cesarean section to total operations performed ratios and emergency herniorrhaphies to all herniorrhaphies performed ratios at Médecins Sans Frontières Operations Centre Brussels projects from 2008 – 2014.**
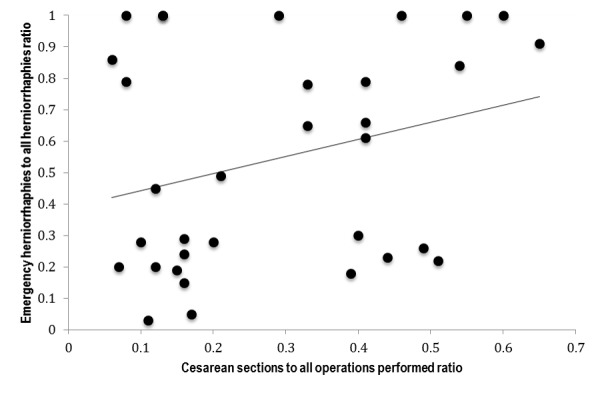

## Discussion

This study aimed to determine CSRs and EHRs from MSF-OCB projects and examine how they correlate as metrics for surgical capacity in low-income countries affected by crisis. Despite operating strictly in areas with known critical deficiencies in surgical capacity further disrupted by crisis, CSRs and EHRs were widely variable and not correlated within respective projects. Therefore, us of these proxies for surgical capacity may not be appropriate in areas affected by crisis.

Though the CSR and EHR seem to be useful for rapid, unrefined surgical capacity assessments in LICs, the ratios may not be accurate reflections of surgical capacity during crisis.^15^^,^ 16 ^,^ 17 This could be due to several reasons: ratios’ dependence on gender, factors unique to crises and changes in surgical condition epidemiology. Since no data exist with which to put this study into context, these potential reasons are considered in detail.

Gender dependence

Since cesarean sections are exclusively performed for women and hernias are significantly more common in males, the CSR and EHR are dependent on usual gender proportions seeking surgical care. Crisis affects health-seeking behavior differently in men and women.^23^^,^ 24 During conflict, males are more likely to be involved in combat, sustain injuries or die and women and children may be less likely to seek or able to access care than during peacetime, even for emergency conditions.^25^^,^ 26 Moreover, conflict and natural disasters cause internal displacement and emigration, exceptionally of women and children.^27^ The resultant unique proportion of adult men left behind may affect the usefulness of the ratios during a crisis.

Factors unique to crisis

Protracted crisis causes changes in birth and fecundity rates, availability of and referral patterns for elective surgery and creates psychological and physical barriers to care.

Birth rates are not constant during times of crisis. While CSR early in crisis may accurately indicate existing surgical capacity at the time of humanitarian assistance deployment, fecundity rates often decrease immediately after crisis ensues and later surpass pre-crisis rates as populations recover.^28^ Resultantly, CSRs may be affected despite there being no change in surgical capacity.

Scheduled clinics and outpatient departments where patients in need of elective surgery are identified are often disrupted during crisis. In addition, standard indications for surgery are less strictly adhered to during crisis to meet other healthcare needs and ensure patient and staff safety.^19^ Therefore, while useful in ‘steady-state’ healthcare systems, these metrics may artificially reflect diminished surgical capacity when there has only been a change in referral patterns or willingness to perform or undergo elective procedures.

The ratios may be dependent on hospitals’ proximity to other surgically capable facilities or reputation of the humanitarian hospital among potential patrons without truly indicating surgical capacity in a defined area. The ratios may vary widely depending on the location of the hospital, such as at the frontlines of conflict or disaster epicenter versus at a location on the periphery caring for those that have fled or been relocated.^19^ Moreover, conflicts and natural disasters often result in psychological or physical barriers to movement that may arise between people needing care and capable hospitals. Thus, there may be a change in the CSR or EHR without a change in ability to provide surgical care.

Variation in surgical condition epidemiology

In addition to the ratios being responsive to changes in surgical capacity, they also represent changes in surgical condition epidemiology. Conflict and natural disasters generate injuries and surgical infections early in the crisis that might lower the CSR or increase the EHR despite no difference in surgical capacity. Subsequently, the ratios would again change as time from the onset of crisis increased as injuries and surgical infections became less common, again without variation in surgical capacity. Therefore, the ratios may be a better reflection of surgical capacity when unmet surgical needs and proportions of specific conditions are relatively constant.

Limitations

Though this study represents the only comparison of CSRs and EHRs published and used a large database with cases from a number of projects and countries, the data must be cautiously interpreted as the negative finding is likely unique to surgical capacity assessment during crises. The operational data used to calculate the ratios does not contain input proxies for surgical capacity (number of staff, theaters, beds, etc.) that may predict one ratio better than the other and demonstrate its usefulness in humanitarian settings. However, the ratios were still not correlated when only first year, fully operational surgical projects were analyzed to demonstrate the baseline surgical need and lack of capacity prior to MSF-OCB’s assistance. Further, to minimize confounding, projects focused only on specialist care or trauma, not offering elective surgery or not fully operational were excluded. The database does not contain information that could further validate one or both of these ratios, such as average wait time for elective surgeries, investment into hospital infrastructure and service delivery or relative changes in patronage. The CSRs were not adjusted for local birth rates, which may affect their reliability. Though national birth rates for the MSF-OCB host countries are published (between 26 – 45%), they do not reflect the disruption of birth rates due to crisis nor the rapidly changing rates over the crises’ duration. Additionally, CSRs from separate projects within the same country often varied considerably. Therefore, no attempt was made to adjust for national birth rates. Despite these limitations and the lack of correlation between the two metrics found, the results are important to humanitarian aid organizations and governments preparing hospitals to care for injuries and surgical conditions during crisis. Though these metrics appear to be useful in steady-state LMIC conditions, other rapid assessment tools for estimating surgical capacity are needed in conflict and disaster settings.

## Conclusion

Surgical humanitarian assistance projects operate in areas with critical surgical capacity deficiencies that are further disrupted by crisis. In order to plan a cost- and clinically-effective response to baseline and acute unmet surgical needs there must be rapid, accurate assessment of surgical capacity. Though CSR and EHR meet these criteria in ‘steady-state’ healthcare systems, they may not be useful during humanitarian crises. Further study of direct surgical capacity improvements and their relationship with these ratios is necessary to document their role in humanitarian settings.

## Competing Interest

The authors have no competing interests.
